# A data-sharing scheme that supports multi-keyword search for electronic medical records

**DOI:** 10.1371/journal.pone.0244979

**Published:** 2021-01-07

**Authors:** Shufen Niu, Wenke Liu, Song Han, Lizhi Fang

**Affiliations:** College of Computer Science and Engineering, Northwest Normal University, Lanzhou, Gansu, China; Victoria University, AUSTRALIA

## Abstract

As cloud storage technology develops, data sharing of cloud-based electronic medical records (EMRs) has become a hot topic in the academia and healthcare sectors. To solve the problem of secure search and sharing of EMR in cloud platforms, an EMR data-sharing scheme supporting multi-keyword search is proposed. The proposed scheme combines searchable encryption and proxy re-encryption technologies to perform keyword search and achieve secure sharing of encrypted EMR. At the same time, the scheme uses a traceable pseudo identity to protect the patient’s private information. Our scheme is proven secure based on the modified Bilinear Diffie-Hellman assumption and Quotient Decisional Bilinear Diffie-Hellman assumption under the random oracle model. The performance of our scheme is evaluated through theoretical analysis and numerical simulation.

## 1 Introduction

An electronic medical record (EMR) is a digital document that contains medical information about a patient; this document is stored, managed, transmitted, and reproduced with electronic devices (computers, health cards, and others) [1]. Compared to the traditional medical record in paper form, EMR has the advantages of large storage capacity, resource saving, convenient query, improved diagnosis and treatment efficiency. With the continuous development of cloud computing, EMR has been rapidly developed, widely used, and gradually improved. A growing number of institutions and individuals use EMR and upload these data to the cloud for storage. Cloud-based systems have more advantages than traditional systems. Users can store and maintain massive data quickly and enjoy high-quality data storage services formed by cloud computing [2].

As a pervasive storage platform, cloud server providers are willing to deploy their EMR storage and application services to cloud servers [3]. Since EMR involves a large amount of patient’s private information, an important task is to prevent the EMR from being leaked by unauthorized users and cloud servers [4, 5]. To ensure data security and user privacy, the data are usually stored in the form of ciphertext in the cloud server, but users encounter the problem of how to search through the ciphertext. Searchable encryption is a cryptographic primitive that has been developed in recent years to assist users when performing keyword search on the ciphertext. This type of encryption fully utilizes abundant computing resources of cloud servers to perform keyword search on the ciphertext [6, 7]. Using searchable encryption technology, users can efficiently search EMR on the cloud server [8]. As the ciphertext of EMR is encrypted with the patient’s public key, the ciphertext can only be decrypted by the patient using the private key, which causes inconvenience in EMR sharing. The re-encryption technology realizes the conversion of the ciphertext [9], which can be converted into the ciphertext that can be decrypted by other users so that the patient’s EMR can be shared.

## 1.1 Related works

To enable precise retrieval of encrypted data, Song et al. [10] first proposed symmetric searchable encryption (SSE) based on stream cipher. However, the key distribution of SSE is difficult, which means that they cannot be applied in many practical applications. To address this issue, Boneh et al. [11] proposed public key encryptions with keyword search scheme (PEKS) and proved its security under the random oracle model. However, a secure channel is necessary to transmit the key in this scenario. Baek et al. [12] first proposed a secure channel free proxy re-encryption with keyword search (SCF-PEKS) model. Xu et al. [13] proposed the concept of public key encryption based on fuzzy keyword search. After the server implements fuzzy keyword search on all ciphertexts, it returns the results to the receiver, and the receiver performs a more accurate keyword search on these results. As searchable encryption provides the capability to query encrypted data with a given keyword, it can be applied to the EMR to protect the patient’s private information, such as information on identity, communication, and medical history. Liu et al. [14] proposed an efficient and secure fine-grained access control scheme, which realized authorized users’ access to the EMR in cloud storage. Li et al. [15] proposed an attribute-based searchable encryption for the EMR system, which reduced the difficulty of key management in a multi-user environment and realized fine-grained access control of EMR by data owners. As certificateless public key cryptography solves the key escrow problem and avoids the use of certificates, Ma et al. [16] proposed a certificateless searchable public key encryption scheme for mobile healthcare systems. In most EMR data-sharing schemes based on searchable encryption, the EMR ciphertext is encrypted by the patient’s public key, so only the patient uses its private key to decrypt. If the patient’s condition is serious, more hospitals are needed for online consultation, and sharing of EMR becomes a problem.

The emergence of proxy re-encryption (PRE) is considered a superior solution to the aforementioned problems. In proxy re-encryption, a semi-trusted agent converts the ciphertext encrypted with the public key of the delegator Alice to the ciphertext encrypted with the public key of the delegatee Bob through the re-encryption key generated by the proxy re-encryption. Shao et al. [17] first proposed a new cryptography primitive called proxy re-encryption with keyword search (PRES) and constructed a bidirectional PRES scheme; the researchers proved the security of this scheme under the random oracle model. Guo et al. [18] proposed the definition and security model of proxy re-encryption with keyword search with a designated tester (dPRES), which can be proved secure under the standard model. Chen et al. [19] proposed the model of limited proxy re-encryption with keyword search (LPREKS) and proved its security under the mBDH assumption and q-DBDHI assumption in the random oracle model.

## 1.2 Our contributions

In this study, we propose an electronic medical record data-sharing scheme that supports multi-keyword search. We simplify the proposal of Chen et al. [19] and apply it to the data sharing of EMR to achieve secure storage, privacy preservation, and secure sharing of EMR. Roughly, the contributions of our scheme are described as follows:

1. We propose a framework for cloud-based EMR sharing with security and privacy preservation for diagnosis improvements in e-Health system. The doctor generates the EMR for the patient and encrypts it using the public key of the patient. The cloud server is responsible for storing the patient’s EMR ciphertext and performs the search operation on EMR.
2. Our scheme can achieve conditional privacy preservation, in which each EMR encrypted by a patient is mapped to a distinct pseudo identity, while a legal hospital can retrieve the real identity of a patient from any pseudo identity. When the true identity of the patient needs to be obtained, the user can send an identity-tracking request to the hospital. After the verification request is legal, the hospital returns the true identity of the patient to the user.
3. We apply the searchable encryption to implement the secure search on the patient’s EMR. The keyword index is stored in the cloud server. When the patient or data user needs to access the patient’s EMR, the patient uses his/her private key and multi-keyword to generate a trapdoor and upload to the cloud server, then the cloud server performs the search operation.
4. In this scheme, EMR can be obtained not only by the patient, but also by the data user, such as medical institution and insurance company. We apply the proxy re-encryption to ensure secure sharing of the patient’s EMR. When the patient wants to access his/her EMR, the patient sends the trapdoor to the cloud server. The cloud server returns EMR ciphertext to the patient. When the data user wants to obtain the patient’s EMR, an authorization request is sent to the patient. After the authorization of the patient, the cloud server generates a re-encryption key to encrypt the EMR ciphertext. After obtaining the re-encryption ciphertext, the data user decrypts it with his/her private key to obtain the patient’s EMR.

## 1.3 Paper organization

The rest of this paper is organized as follows. In section 2, we present some preliminaries. In section 3, we introduce the system architecture, threat model and design goals, and algorithm model of our scheme. In section 4, we provide an overview of our scheme and describe the scheme in detail. Section 5 provides the security analysis, including the achieving goals and security proof of our scheme. In section 6, we compare the proposed scheme with relevant schemes through theoretical analysis and numerical simulation. Finally, we conclude the paper in section 7.

## 2 Preliminaries

### 2.1 Bilinear map

Let *G*_1_ and *G*_2_ be two cyclic groups of a large prime *q*. Let *g* be a generator of *G*_1_. A bilinear pairing *e* is a function defined by *e*: *G*_1_ × *G*_1_ → *G*_2_ if the function *e* satisfies the following properties:

1. Bilinearity: For any $a,b\in Z_{q}^{*}$, *e*(*g*^*a*^, *g*^*b*^) = *e*(*g*, *g*)^*ab*^.
2. Non-degeneracy: *e*(*g*, *g*) ≠ 1.
3. Computablility: *e*(*g*, *g*) can be efficiently computed.

### 2.2 Hardness assumptions

Let *G*_1_ be a cyclic group of a large prime *q* with a generator *g*. The following assumptions hold in our scheme.

*Definition 1*. *(Modified Bilinear Diffie-Hellman (mBDH) Problem)* [20]. *Given* (*g*, *g*^*a*^, *g*^*b*^, *g*^*c*^) ∈ *G*_1_ for $a,b,c\in Z_{q}^{*}$, the mBDH problem is to compute *e*(*g*, *g*)^*ab*/*c*^.

**mBDH assumption**. We say the mBDH assumption holds if no probabilistic polynomial-time algorithm can solve the mBDH problem with a non-negligible advantage.

*Definition 2. (Quotient Decisional Bilinear Diffie-Hellman (QDBDH) Problem)* [21]. *Given* (*g*, *g*^*a*^, *g*^*b*^)∈*G*_1_ and *Q* ∈ *G*_2_ for $a,b\in Z_{q}^{*},g\in G_{1}$, the QDBDH problem is to determine whether *Q* = *e*(*g*, *g*)^*a*/*b*^ or not.

**QDBDH assumption**. We say the QDBDH assumption holds if no probabilistic polynomial-time algorithm can solve the QDBDH problem with a non-negligible advantage.

## 3 System model

In this section, we present an architecture for the EMR system. Moreover, we consider several threats and propose several design goals.

### 3.1 System architecture

**Fig 1:**
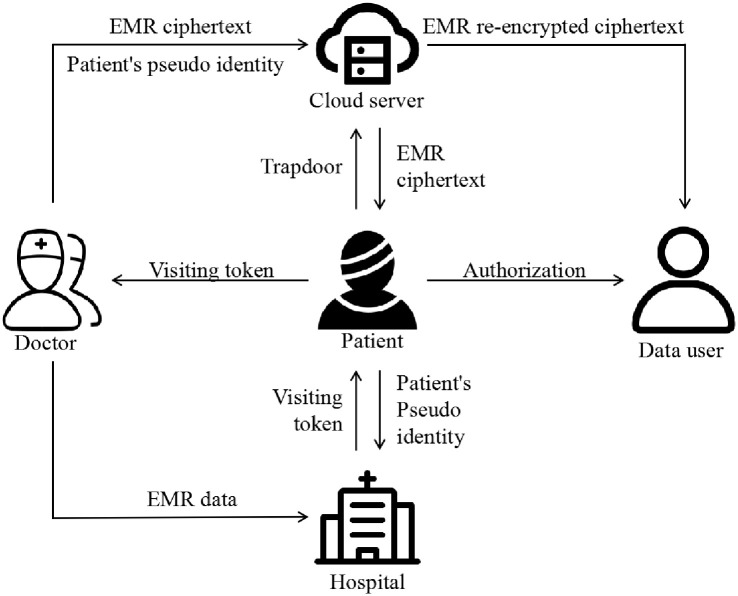
System model.

As shown in Fig 1, five entities are involved in this system: patients, doctor, hospital, cloud server, and data users.

**Patient**. A patient is an entity who needs medical assistance. The patient first needs to register at the hospital to obtain his/her visiting token. When a patient visits a doctor for treatment, his/her health information is generated by the doctor. When the patient’s EMR is needed, he/she can access the EMR by sending the trapdoor to the cloud server. In addition, the patient calculates a pseudo-identity for himself/herself and sends it to the hospital.

**Doctor**. The doctor is an entity responsible for generating the EMR for the patient and uploading them to the hospital. The doctor is also responsible for encrypting the EMR with the patient’s public key and sends the ciphertext to the cloud server. When the doctor wants to obtain the patient’s historical EMR, the doctor sends a request to the patient. After receiving the EMR from the cloud server, the patient shows the EMR to the doctor.

**Hospital**. A hospital is an entity that is responsible for generating a visiting token with value *τ* for the patient and sending the token to the cloud server. The hospital is also responsible for calculating the true identity of the patient required by the data user.

**Cloud server**. The cloud server is an entity that takes responsibility for storing the patient’s encrypted EMR ciphertext and providing the function of searching EMR. After receiving the trapdoor from the patient, the cloud server performs the search operation on EMR. The cloud server generates the re-encryption key by interacting with data users and patients. Then, the cloud server re-encrypts the EMR ciphertext using the re-encryption key and sends the re-encryption ciphertext to the data user.

**Data user**. In our scheme, the data user refers to the user authorized by the patient who wants to use the patient’s EMR. For example, if a patient’s condition is complicated, multiple experts are needed for consultation, and the experts come from different hospitals. After interacting with the patient and the cloud server, the data user receives the re-encryption ciphertext sent by the cloud server. The data user can decrypt it using his/her private key.

### 3.2 Threat model and design goals

In this study, we consider a semi-trust server that has been widely utilized in existing work. Specifically, the server honestly searches information for the benefit of patients, but curiously learns the underlying meaning of the sender’s EMR. In addition, malicious outside attackers may intercept and analyze the information transferred in the public channel. Based on the preceding system architecture and threat model, the design goals of our scheme are as follows:

1. Data confidentiality and integrity. Whether the EMR is stored on the hospital server or transmitted through the public channel, no entity can retrieve or modify the EMR data.
2. Access control. The EMR data belongs to the patients who can control data access. In other words, only authorized users have the right to access the data. Simultaneously, data access activities should always be carried out with the participation and monitoring of patients and hospitals.
3. Secure search. When the doctor wants to access the patient’s history EMR to improve diagnosis, the patient generates a trapdoor to search the EMR. During the process, only patients can generate the trapdoor. Moreover, the pseudo-identity of the patient is used in the search process, so the eavesdropper cannot deduce the real identity of the patient.
4. Privacy preservation. As the EMR data contains privacy-sensitive information of the patient, the patient’s identity must be kept secret.

### 3.3 Algorithm description

The proposed scheme is composed of nine polynomial-time algorithms:

**Setup**(1^λ^) → *PP*: The algorithm takes a security parameter 1^λ^ as input, and outputs the public parameters *PP*.

**KeyGen**(*PP*) → (*pk*, *sk*): Given the public parameters *PP*, the algorithm outputs a public/private key pair (*pk*, *sk*).

**Enc**(*pk_P_*, *W*, *M*) → *C*: The algorithm inputs a public key of user *P*, an electronic medical record *M*, a keyword set *W* = (*w*_1_, ⋯, *w*_*n*_), outputs an original ciphertext *C*.

$\text{Trapdoor}\left(sk_{P},Q\right)\to T_{w^{{}^{\prime }}}:$ Takes a private key of user *P* and a query keyword set

$Q=(w_{1}^{{}^{\prime }},\cdots ,w_{n}^{{}^{\prime }})$ as input, the algorithm outputs a keyword trapdoor set

$T_{w^{{}^{\prime }}}=\left(T_{w_{1}^{{}^{\prime }}},\cdots ,T_{w_{n}^{{}^{\prime }}}\right)$.

$\text{Search}(T_{w^{{}^{\prime }}},C)\to 1\;or\;0:$ Given a trapdoor set $T_{w^{{}^{\prime }}}=\left(T_{w_{1}^{{}^{\prime }}},\cdots ,T_{w_{n}^{{}^{\prime }}}\right)$ and a ciphertext *C*, the algorithm outputs 1 if $w_{i}^{{}^{\prime }}=w_{i}$ for 1 ≤ *i* ≤ *n*, or 0 otherwise.

**Dec_1_**(*sk_P_*, *C*) → *M*: The algorithm takes a private key of user *P* and an original ciphertext *C*, and output a record *M* if each input parameter is correct.

**ReKeyGen**(*sk_P_*, *sk_R_*) → *rk*_*P*→*R*_: Given user *P*’s private key *sk*_*P*_ and user *R*’s private key *sk*_*R*_, the algorithm outputs a re-encryption key *rk*_*P*→*R*_. This process is performed by user *P*, user *R* and the cloud server.

**ReEnc**(*rk*_*P*→*R*_, *C*) → *C*′: Takes a re-encryption key *rk*_*P*→*R*_ from user *P* to user *R* and an original ciphertext *C* for user *P*, the algorithm converts the ciphertext *C* to *C*′ for user *R*.

**Dec**(*sk_R_*, *C*′) → *M*: The algorithm takes a private key of user *R* and a re-encryption ciphertext *C*′, and output a record *M* if each input parameter is correct.

## 4 EMR sharing

### 4.1 Overview of scheme

Without loss of generality, we assume that a patient *P* registers to a hospital for medical assistance, and the hospital generates a visiting token *τ* for the patient and sends it to the patient. Here, *τ* works as the authorization for the doctor to generate EMR for the patient *P*. Meanwhile, the patient *P* computes a pseudo identity *ID*_*P*_ for himself/herself and returns it to the hospital. The hospital packs the tuple (*ID*_*P*_, *τ*) and sends it to the cloud server. After the patient *P* physically visits the doctor, he/she provides *τ* to the doctor as accordance for generating his/her EMR. We assume that the doctor generates health record *M* for the patient *P* by the interaction. To safely store the data with interoperability, the doctor extracts a keyword set *W* = (*w*_1_, ⋯, *w*_*n*_) for the EMR. Then, the doctor encrypts *M* and *W* with the patient’s public key *pk*_*P*_. The ciphertext *C* = (*C*_*M*_, *C*_*W*_) is stored in the cloud server, where *C*_*M*_ is the ciphertext of EMR *M* and *C*_*W*_ is the ciphertext of keyword set *W*.

When the patient *P* visits another doctor in a different hospital, the doctor may think it is necessary to know the patient’s history health record. The patient *P* can send an access request that includes keyword trapdoor to the cloud server. If the access request is valid, the cloud server sends the patient *P* the ciphertext *C*_*M*_. The patient *P* can decrypt *C*_*M*_ with his/her private key to obtain the health record *M*. Then, the patient shows it to the doctor.

If the data user *R* wants to access the EMR of patient *P*, then he/she sends an interactive request to the patient and the cloud server. After the interaction, the cloud server generates a re-encryption key. The cloud server uses this key to re-encrypt the EMR ciphertext and obtains the re-encryption ciphertext. Then, the cloud server sent it to the data user *R*. The data user *R* uses his own private key to decrypt the re-encryption ciphertext. If the data user wants to obtain the true identity of the patient *P*, he/she can send a request to the hospital.

### 4.2 Our scheme

In this section, we introduce the details of our proposed scheme. The entities in our scheme involved at least one of the algorithms mentioned in “algorithm definition”. Roughly, our proposed scheme is composed of four main phases: initialization, data processing, search, and record retrieval.

#### Phase 1: Initialization

In this phase, the system generates the public parameter *PP* by operating the algorithm *Setup*(1^λ^), where 1^λ^ is the security parameter. All the patients *P*, doctors *D*, and data users *R* generate their private and public keys by running the algorithm *KeyGen*(*PP*).

**Setup**(1^λ^): Select two bilinear groups (*G*_1_, *G*_2_) of prime order *q* and a bilinear map *e*. Pick *g* as a generator of *G*_1_ and set *Z* = *e*(*g*, *g*). Select four hash functions *H*_1_: *G*_1_ → {0, 1}*, *H*_2_: {0, 1}* → *G*_1_, *H*_3_: *G*_2_ → {0, 1}^*log*2*q*^, *H*_4_: *G*_2_ → {0, 1}*. Thus, the public parameter can be denoted as *PP* = {*G*_1_, *G*_2_, *g*, *q*, *e*, *Z*, *H*_1_, *H*_2_, *H*_3_, *H*_4_}.
**KeyGen**(*PP*): Each patient *P* randomly selects a secret value $p\in Z_{q}^{*}$ as its private key *sk*_*P*_ and computes the public key *pk*_*P*_ = *g*^*p*^. Each doctor *D* randomly chooses a secret value $d\in Z_{q}^{*}$ as its private key *sk*_*D*_ and computes the public key *pk*_*D*_ = *g*^*d*^. Each data user *R* randomly selects a secret value $r\in Z_{q}^{*}$ as its private key *sk*_*R*_ and computes the public key *pk*_*R*_ = *g*^*r*^.

When the patient *P* registers at the hospital, the hospital randomly selects *β* ∈ {0, 1}* and computes *τ* = *g*^1/*β*^. Then, the hospital sends the token *τ* to the patient *P* securely. Meanwhile, the patient randomly selects $s\in Z_{q}^{*}$ and computes *S* = *g*^*s*^. Thereafter, the patient calculates his/her pseudo identity *ID*_*P*_ = *RID*_*P*_ ⊕ *H*_1_(*τ*^*s*^) where *RID*_*P*_ is the real identity of the patient *P*. The patient *P* returns the tuple (*τ*, *S*, *ID*_*P*_) to the hospital. The hospital chooses a doctor *D* for the patient and sends the tuple to the cloud server with the doctor.

#### Phase 2: Data encryption and storage

As a patient *P* sees a doctor *D* for medical assistance, he/she shows the doctor token *τ*, which works as a proof of the patient’s authorization to the doctor for generating his/her EMR. After interaction with the patient *P*, the doctor *D* generates health record *M* ∈ *G*_2_ and extracts a keyword set *W* = (*w*_1_, ⋯, *w*_*n*_) from the record. Then, the doctor stores *M* in the hospital and encrypts *M* and *W* with the patient’s public key *pk*_*P*_ by operating the algorithm *Enc*(*pk*_*P*_, *W*, *M*).

**Enc**(*pk_P_*, *W*, *M*): The doctor randomly selects a value $k\in Z_{q}^{*}$ and computes *C*_1_ = *M* ⋅ *Z*^*k*^, $C_{2}=pk_{P}^{k}$, *C*_3_ = *H*_4_(*C*_1_), $t=e{\left(\sum_{i=1}^{n}H_{2}\left(w_{i}\right),g\right)}^{k}$ for 1 ≤ *i* ≤ *n*.

The output of encryption algorithm is *C* = (*C*_*M*_, *C*_*W*_), where *C*_*M*_ = (*C*_1_, *C*_2_) and *C*_*W*_ = (*t*, *H*_3_(*t*)). Here, *C*_*M*_ is the record ciphertext and *C*_*W*_ is the keyword index. The doctor sends the ciphertext *C* and the patient’s pseudo identity *ID*_*P*_ to the cloud server. To match the patient’s token in the cloud server, the doctor performs the following operations:

- Randomly chooses value $a\in Z_{q}^{*}$ and computes $\alpha =g^{\frac{a+d}{H_{1}\left(\tau \right)}}$, $\tau^{{}^{\prime }}=H_{1}\left(g^{a}\right)⊕H_{1}\left(\tau \right)$.

The doctor sends (*α*, *τ*′) to the cloud server. Then, the cloud server checks whether the equation *H*_1_(τ*) = *H*_1_(*τ*) holds or not, where $H_{1}\left(\tau^{*}\right)=H_{1}\left(\alpha^{H_{1}\left(\tau \right)}\cdot pk_{D}^{-1}\right)⊕\tau^{{}^{\prime }}$. If the equality holds, the EMR ciphertext *C* successfully matches the token *τ* of the patient *P*. The cloud server stores the ciphertext *C* and *ID*_*P*_ together.

**Correctness**:

$$\begin{array}{c} \begin{array}{cc} H_{1}\left(\tau^{*}\right) & =H_{1}\left(\alpha^{H_{1}\left(\tau \right)}\cdot pk_{D}^{-1}\right)⊕\tau^{{}^{\prime }}\\ & =H_{1}\left({\left(g^{\frac{a+d}{H_{1}\left(\tau \right)}}\right)}^{H_{1}\left(\tau \right)}\cdot g^{-d}\right)⊕H_{1}\left(g^{a}\right)⊕H_{1}\left(\tau \right)\\ & =H_{1}\left(g^{a}\right)⊕H_{1}\left(g^{a}\right)⊕H_{1}\left(\tau \right)\\ & =H_{1}\left(\tau \right) \end{array} \end{array}$$

#### Phase 3: Search

This phase is divided into two steps: trapdoor generation and test. On another day, the patient may visit another doctor in a different hospital. During the interaction process of the doctor and the patient, the doctor may find that it is necessary to access the patient’s history record for a more accurate diagnosis. To search over the encrypted record *C*, the patient *P* needs to compute the trapdoor set for a query keyword set $Q=(w_{1}^{{}^{\prime }},\cdots ,w_{n}^{{}^{\prime }})$ by invoking the algorithm *Trapdoor*(*sk*_*P*_, *Q*).

**Trapdoor**(*sk_P_*, *Q*): The patient *P* computes $T_{w^{{}^{\prime }}}=\left\{T_{w_{i}^{{}^{\prime }}}=H_{2}{\left(w_{i}^{{}^{\prime }}\right)}^{\frac{H_{1}(\tau }{p})},1\leq i\leq n\right\}$.

Meanwhile, the patient *P* sets an effective access time *tr* for this request [22], and then sends a tuple (*tr*, *T*_*w*′_) to the cloud server.

The cloud server checks the validity of *tr* after receiving the tuple. If *tr* is not effective, the message is ignored. Otherwise, the cloud server performs *Search* $\left(T_{w^{{}^{\prime }}},C\right)$ to check whether the encrypted record *C* involves the keyword set *Q*. Precisely, for each *w*_*i*_ in *Q*, the cloud server checks whether the equation $H_{3}\left(\prod_{i=1}^{n}e\left(C_{2},T_{w_{i}^{{}^{\prime }}}\right)\right)=H_{3}\left(t^{H_{1}\left(\tau \right)}\right)$ holds or not. If the equality holds, then the cloud server sends EMR ciphertext *C*_*M*_ to the patient *P*. Otherwise, it sends ⊥.

**Correctness**:

$$\begin{array}{c} \begin{array}{cc} H_{3}\left(\prod_{i=1}^{n}e\left(C_{2},T_{w_{i}^{{}^{\prime }}}\right)\right) & =H_{3}\left(\prod_{i=1}^{n}e\left(g^{p\cdot k},H_{2}{\left(w_{i}^{{}^{\prime }}\right)}^{\frac{H_{1}\left(\tau \right)}{p}}\right)\right)\\ & =H_{3}\left(\prod_{i=1}^{n}e{\left(g,H_{2}\left(w_{i}^{{}^{\prime }}\right)\right)}^{k\cdot H_{1}\left(\tau \right)}\right)\\ & =H_{3}\left(e{\left(g,\sum_{i=1}^{n}H_{2}\left(w_{i}^{{}^{\prime }}\right)\right)}^{k\cdot H_{1}\left(\tau \right)}\right)\\ & =H_{3}\left(t^{H_{1}\left(\tau \right)}\right) \end{array} \end{array}$$

#### Phase 4: Record retrieval

This phase involves two cases: the patient decrypts EMR and the data user decrypts EMR.

**Case 1**: The patient decrypts EMR.

Upon receiving EMR ciphertext *C*_*M*_ from the cloud server, the patient *P* decrypts the ciphertext *C*_*M*_ to retrieve the record *M* by invoking the algorithm *Dec*_1_(*sk*_*P*_, *C*).

**Dec**(*sk_P_*, *C*): The patient *P* calculates $M=\frac{C_{1}}{e{\left(g,C_{2}\right)}^{\frac{1}{p}}}$.

After obtaining the EMR *M*, the patient *P* shows it to the doctor.

**Correctness**:

$$\begin{array}{c} \begin{array}{cc} \frac{C_{1}}{e{\left(g,C_{2}\right)}^{\frac{1}{p}}} & =\frac{M\cdot Z^{k}}{e{\left(g,g^{p\cdot k}\right)}^{\frac{1}{p}}}\\ & =\frac{M\cdot e{\left(g,g\right)}^{k}}{e(g,g^{k})}\\ & =M \end{array} \end{array}$$

**Case 2**: The data user decrypts EMR.

To obtain the patient *P*’s EMR, the data user *R* first requests the patient *P* and cloud servers to interact with him/her. The cloud server generates the re-encryption key by running the algorithm *ReKeyGen*(*sk*_*P*_, *sk*_*R*_). More precisely, the re-encryption key is generated by the following steps:

- The patient *P* randomly chooses value $j\in Z_{q}^{*}$. Then, the patient *P* sends *j* to the cloud server and *sk*_*P*_ ⋅ *j* to the data user *R*.
- After receiving *sk*_*P*_ ⋅ *j* from the patient *P*, the data user *R* sends *sk*_*R*_/(*sk*_*P*_ ⋅ *j*) to the cloud server.
- Finally, the cloud server computes the re-encryption key *rk*_*P*→*R*_ = *r*/*p*.

Then, the cloud server re-encrypts the EMR ciphertext *C*_*M*_ with *rk*_*P*→*R*_ to generate the re-encryption ciphertext $C_{M}^{{}^{\prime }}$ for the data user *R* by running the algorithm *ReEnc*(*rk*_*P*→*R*_, *C*).

**ReEnc**(*rk*_*P*→*R*_, *C*): The cloud server computes $C_{1}^{{}^{\prime }}=C_{1}$, $C_{2}^{{}^{\prime }}=C_{2}^{rk_{P\to R}\cdot H_{4}\left(C_{1}^{{}^{\prime }}\right)}$ $=g^{r\cdot k\cdot H_{4}\left(C_{1}^{{}^{\prime }}\right)}$, $C_{3}^{{}^{\prime }}=C_{3}$.

The cloud server sets the re-encryption ciphertext $C_{M}^{{}^{\prime }}=\left(C_{1}^{{}^{\prime }},C_{2}^{{}^{\prime }},C_{3}^{{}^{\prime }}\right)$ and sends it to the data user *R*. After receiving EMR re-encryption ciphertext $C_{M}^{{}^{\prime }}$ from the cloud server, the data user *R* decrypts it to retrieve the record *M* by invoking the algorithm *Dec*_2_(*sk*_*R*_, *C*).

**Dec**(*sk_R_*, *C*): The data user *R* calculates $M=C_{1}^{{}^{\prime }}/e{\left(g,C_{2}^{{}^{\prime }}\right)}^{1/(r\cdot C_{3}^{{}^{\prime }})}$.

When the real identity of the patient *P* needs to be obtained for treatment or medical insurance purposes, the data user *R* sends a request to the hospital. The hospital obtains the true identity of the patient *P* by calculating *RID*_*P*_ = *ID*_*P*_ ⊕ *H*_4_(*S*^1/*β*^) and returns it to the data user *R*. In our scheme, only the hospital system knows the *β* value, so only the hospital can extract the real identity of the patient.

**Correctness**:

$$\begin{array}{c} \begin{array}{cc} \frac{C_{1}^{{}^{\prime }}}{e{\left(g,C_{2}^{{}^{\prime }}\right)}^{\frac{1}{r\cdot C_{3}^{{}^{\prime }}}}} & =\frac{M\cdot Z^{k}}{e{\left(g,g^{r\cdot k\cdot H_{4}\left(C_{1}\right)}\right)}^{\frac{1}{r\cdot H_{4}\left(C_{1}\right)}}}\\ & =\frac{M\cdot e{\left(g,g\right)}^{k}}{e{\left(g,g\right)}^{k}}\\ & =M \end{array} \end{array}$$

## 5 Security analysis

### 5.1 Achieving goals

In this section, we illustrate how the proposed scheme can effectively achieves the design goals presented in “System Model”.

**The proposed scheme achieves data confidentiality and integrity**. The EMR data are encrypted before being outsourced to the hospital server. The doctor uses the patient’s public key to encrypt the EMR. On the one hand, the patient uses his/her private key to decrypt the EMR ciphertext; on the other hand, the data user authorized by the patient uses his/her private key to decrypt the EMR re-encryption ciphertext.

**The proposed scheme achieves access control**. As mentioned in phase 4, if the data user wants to access the patient’s EMR, he/she first sends an authorization request to the patient. After the patient agrees, the cloud server generates a re-encryption key. The cloud server re-encrypts the EMR ciphertext with it to generate the re-encryption ciphertext that the data user can decrypt with his/her private key.

**The proposed scheme achieves secure search**. In phase 2 of our scheme, the EMR is encrypted with keyword search. In phase 3, the patient generates the trapdoor set to search his/her history health record to improve the doctor’s diagnosis of the patient. In this scenario, the keyword trapdoor $T_{w^{{}^{\prime }}}=\left\{T_{w_{i}^{{}^{\prime }}}=H_{2}{\left(w_{i}^{{}^{\prime }}\right)}^{H_{1}\left(\tau \right)/p},1\leq i\leq n\right\}$ contains the patient’s private key, so only the patient can generate the trapdoor and perform search on EMR.

### 5.2 Security proof

As the data used by the patient is similar to that of the data user, we only demonstrate the safety of data used by data users.

**Theorem 1**. *Our scheme is IND-CKA secure in the random oracle model, if mBDH assumption holds in G*_1_ and *G*_*T*_.

**Proof**. We assume the existence of a polynomial-time adversary *A*_1_ with non-negligible advantage *ϵ*(*k*) in attacking the privacy for keywords of our scheme, where *ϵ*(*k*) is a negligible function in the security parameter *k*. We construct a simulator *B* that can compute the solution of the mBDH problem.

Let (*g*, *g*^*α*^, *g*^*β*^, *g*^*γ*^ ∈ *G*_1_) be an instance of the mBDH problem, where *g* is the generator of *G*_1_ and $\alpha ,\beta ,\gamma \in Z_{q}^{*}$ are uniformly random choices. The goal of *B* is to output *e*(*g*, *g*)^*αβ*/*γ*^ ∈ *G*_2_ by interacting with *A*_1_ as follows:

***H*_1_ query**: *B* maintains an empty-initial table $H_{1}^{list}$. Input *w* in the hash function *H*_1_, and *B* checks $H_{1}^{list}$. If <*w*_*i*_, *h*_*i*_, *a*_*i*_, *c*_*i*_> exists in $H_{1}^{list}$, then *B* returns *H*_1_(*w*_*i*_) = *h*_*i*_. Otherwise, *B* generates a random coin *c*_*i*_ such that *pr*[*ci* = 0] = 1/(*q*_*T*_ + 1), where *c*_*i*_ ∈ {0, 1}, *q*_*T*_ is the maximum number of Trapdoor queries. *B* selects a random number $a_{i}\in Z_{q}^{*}$. If *c*_*i*_ = 0, *B* returns $h_{i}={\left(g^{\alpha }\right)}^{a_{i}}$ to *A*_1_; if *c*_*i*_ = 1, *B* returns $h_{i}={\left(g^{\gamma }\right)}^{a_{i}}$ to *A*_1_. Thereafter, *B* adds <*w*_*i*_, *h*_*i*_, *a*_*i*_, *c*_*i*_> to $H_{1}^{list}$.

***H*_2_ query**: *B* maintains an empty-initial table $H_{2}^{list}$. Upon receiving *H*_2_ query about *t*′ ∈ *G*_2_ from *A*_1_, *B* checks $H_{2}^{list}$. If *t*′ already exists in $H_{2}^{list}$, *B* returns *V* to *A*_1_. Otherwise, *B* selects a value *V* ∈ {0, 1}^*log*_2_*q*^ randomly and adds <*t*′, *V*> to $H_{2}^{list}$ by setting $H_{2}\left(t^{{}^{\prime }}\right)=V$.

**Phase 1**. *A*_1_ makes several queries.

**Uncorrupted key query**: On input an index *i*, *B* selects $x_{i}\in Z_{q}^{*}$ randomly and outputs the public key $pk_{i}={\left(g^{\gamma }\right)}^{x_{i}}$. Thus, the private key is defined as *sk*_*i*_ = *γx*_*i*_ implicitly. *B* adds <*i*, *pk*_*i*_, *x*_*i*_> to *L*_*U*_.

**Corrupted key query**: On input an index *i*, *B* selects $x_{i}\in Z_{q}^{*}$ randomly and outputs the public key $pk_{i}=g^{x_{i}}$. Thus, the private key is defined as *sk*_*i*_ = *x*_*i*_ implicitly. *B* adds <*i*, *pk*_*i*_, *x*_*i*_> to *L*_*U*_.

**Trapdoor query**: When *A*_1_ makes a trapdoor query on the keyword *w*_*i*_, *B* responds as follows:

- *B* recovers <*w*_*i*_, *h*_*i*_, *a*_*i*_, *c*_*i*_>,<*i*, *pk*_*i*_, *x*_*i*_>,<*i*, *pk*_*i*_, *x*_*i*_> from $H_{1}^{list}$, *L*_*U*_, *L*_*C*_, respectively.
- If *c*_*i*_ = 0, *B* aborts. Otherwise, *B* computes $h_{i}={\left(g^{\gamma }\right)}^{a_{i}}$ when *c*_*i*_ = 1.
- If *i* ∈ *L*_*C*_, *B* computes $T_{i}=h_{i}^{N/x_{i}}={\left(g^{\gamma }\right)}^{Na_{i}/x_{i}}$; if *i* ∈ *L*_*C*_, *B* computes $T_{i}=h_{i}^{N/(\gamma x_{i})}={\left({\left(g^{\gamma }\right)}^{a_{i}}\right)}^{N/(\gamma x_{i})}=g^{Na_{i}/x_{i}}$. Then, *T*_*i*_ is the trapdoor for keyword *w*_*i*_ and *B* returns *T*_*i*_ to *A*_1_.

**Re-encryption key query**: When *A*_1_ asks *B* about the re-encryption key *rk*_*i*→*j*_ for two public keys *pk*_*i*_, *pk*_*j*_, *B* responds as follows:

- If neither *pk*_*i*_ nor *pk*_*j*_ belongs to *L*_*C*_, *B* aborts.
- Otherwise, *B* returns *rk*_*i*→*j*_ = *x*_*j*_/*x*_*i*_ to *A*_1_.

**Challenge**: Eventually, *A*_1_ issues a challenge on two keywords *w*_0_, *w*_1_, a message *m*, and a public key *pk*_*i*_. If *pk*_*i*_ belongs to *L*_*C*_, then *B* aborts. Otherwise, *B* performs as follows:

- *B* conducts two *H*_1_ queries to obtain *h*_0_, *h*_1_ ∈ *G*_1_ such that *H*_1_(*w*_0_) = *h*_0_, *H*_1_(*w*_1_) = *h*_1_. If both *c*_0_ = 1 and *c*_1_ = 1 hold, then *B* aborts.
- Otherwise, at least one of *c*_0_ and *c*_1_ is equal to 0. Then *B* randomly picks *b* ∈ {0, 1} so that *c*_*b*_ = 0.
- *B* returns $C_{b}^{{}^{\prime }}=\left(g^{\beta x_{i},V}\right)$ to *A*_1_, where *V* ∈ {0, 1}^*log*_2_*q*^.
- *B* implicitly defines $pk_{i}^{\beta /\gamma }={\left({\left(g^{\gamma }\right)}^{x_{i}}\right)}^{\beta /\gamma }$ and $V=H_{2}\left(e{\left(H_{1}\left(w_{b}\right),g\right)}^{\beta /\gamma }\right)=H_{2}\left(e{\left(g^{\alpha a_{b}},g\right)}^{\beta /\gamma }\right)=H_{2}\left(e\left({\left(g^{\alpha a_{b}}\right)}^{1/\gamma x_{i}},g^{\beta x_{i}}\right)\right)=H_{2}\left(e{\left(g,g\right)}^{\beta \alpha a_{b}/\gamma }\right)$.

**Phase 2**. *A*_1_ can continue to issue several queries as in phase 1 on keyword *w*_*i*_, where *w*_*i*_ ≠ *w*_0_ and *w*_*i*_ ≠ *w*_1_.

**Guess**: Finally, *A*_1_ outputs its guess *b*′ ∈ {0, 1} to check whether the challenge ciphertext $C_{b}^{{}^{\prime }}$ is the result of keyword *w*_0_ or *w*_1_. Then *B* chooses the pair (*t*′, *V*) from $H_{2}^{list}$ and outputs ${\left(t^{{}^{\prime }}\right)}^{1/a_{b}}$ as its guess to *e*(*g*, *g*)^*βα*/*γ*^.

**Theorem 2**. *Our scheme is IND-CPA secure in the random oracle model, if QDBDH assumption holds in G*_1_ and *G*_*T*_.

**Proof**. We assume the existence of a polynomial-time adversary *A*_2_ with non-negligible advantage *ϵ*(*k*) in attacking our scheme, where *ϵ*(*k*) is a negligible function in the security parameter *k*. We construct a simulator *B* that can compute the solution of the QDBDH problem.

Let (*g*, *g*^*a*^, *g*^*b*^ ∈ *G*_1_) be an instance of the mBDH problem, where *g* is the generator of *G*_1_ and $a,b\in Z_{q}^{*}$ are uniformly random choices. The goal of *B* is to output *e*(*g*, *g*)^*a*/*b*^ ∈ *G*_2_ by interacting with *A*_2_ as follows:

***H*_1_ query**: *B* maintains an empty-initial table $H_{1}^{list}$. Once receiving *H*_1_ query about $w\in Z_{q}^{*}$ from *A*_2_, *B* checks $H_{1}^{list}$. If *w* already exists in $H_{1}^{list}$, *B* returns *h* to *A*_2_. Otherwise, *B* selects a value *h* ∈ {0, 1}^*log*_2_*q*^ randomly and adds <*w*, *h*> to $H_{1}^{list}$ by setting *H*_1_(*w*) = *h*.

***H*_2_ query**: *B* maintains an empty-initial table $H_{2}^{list}$. Once receiving *H*_2_ query about *t*′ ∈ *G*_2_ from *A*_2_, *B* checks $H_{2}^{list}$. If *t*′ already exists in $H_{2}^{list}$, *B* returns *V* to *A*_2_. Otherwise, *B* selects a value *V* ∈ {0, 1}^*log*_2_*q*^ randomly and adds <*t*′, *V*> to $H_{2}^{list}$ by setting $H_{2}\left(t^{{}^{\prime }}\right)=V$.

**Phase 1**. *A*_2_ makes several queries.

**Public key query**: *B* generates a random coin *c* ∈ {0, 1}. If *c*_*i*_ = 1, *B* selects a random value $x_{i}\in Z_{q}^{*}$ and outputs the public key $pk_{i}=g^{ax_{i}}$. Otherwise, *B* outputs the public key $pk_{i}=g^{x_{i}}$ and adds <*c*_*i*_, *pk*_*i*_, *x*_*i*_> to table *L*_*C*_, where the private key is implicitly defined as *sk*_*i*_ = *x*_*i*_.

**Private key query**: *B* recovers <*c*_*i*_, *pk*_*i*_, *x*_*i*_> from *L*_*C*_. If *c*_*i*_ = 0, the private key *sk*_*d*_ = *x*_*d*_ is returned to *A*_2_. Otherwise, it aborts.

**Re-encryption key query**: The adversary *A*_2_ can adaptively ask *B* for the re-encryption key *rk*_*i*→*j*_ for any two public keys *pk*_*i*_, *pk*_*j*_ and *B* generates the re-encryption key as follows:

- If *c*_*i*_ = 1 and *c*_*j*_ = 1, *B* aborts.
- Otherwise, *B* responds *rk*_*i*→*j*_ = *x*_*j*_/*x*_*i*_ to *A*_2_.

**Re-encryption query**: Based on the result of re-encryption query, *B* obtains the re-encryption ciphertext through the re-encryption algorithm and returns it to *A*_2_.

**Decryption query**: After obtaining the re-encryption ciphertext $C_{m}^{{}^{\prime }}=\left(C_{1}^{{}^{\prime }},C_{2}^{{}^{\prime }},C_{3}^{{}^{\prime }}\right)$, B recovers <*c*_*i*_, *pk*_*i*_, *x*_*i*_> associated with the data user from *L*_*C*_. If *c*_*i*_ = 0, *B* set the message $m=\frac{C_{1}^{{}^{\prime }}}{e{\left(g,C_{2}^{{}^{\prime }}\right)}^{1/(sk\cdot C_{3}^{{}^{\prime }})}}$. Otherwise, *B* sets the message $m=\frac{C_{1}^{{}^{\prime }}}{e{\left(g^{a},C_{2}^{{}^{\prime }}\right)}^{1/(sk\cdot C_{3}^{{}^{\prime }})}}$.

**Challenge**: Eventually, *A*_2_ issues a challenge on two messages *m*_0_, *m*_1_ and a public key *pk*_*i*_. *B* recovers the tuple <*c*_*i*_, *pk*_*i*_, *x*_*i*_> from *L*_*C*_. If *c* = 1, then *B* reports failure and aborts. Otherwise, *B* randomly selects *δ* ∈ {0, 1} and sets the challenge ciphertext $C_{\delta }^{{}^{\prime }}$ as follows:

$$C_{1}^{*}=m\;\cdot \;T,C_{2}^{*}={\left(pk^{*}\right)}^{a/b}$$

**Phase 2**. *A*_2_ can continue to issue several queries as in phase 1 on message *m*_*i*_, where *m*_*i*_ ≠ *m*_0_ and *m*_*i*_ ≠ *m*_1_.

**Guess**: Finally, *A*_2_ outputs its guess *b*′ ∈ {0, 1} to check whether the challenge ciphertext $C_{\delta }^{{}^{\prime }}$ is the result of message *m*_0_ or *m*_1_. If *c*_*δ*_ = 1, then the ciphertext $C_{1}^{*}=m\cdot T=m\cdot e{\left(g,g\right)}^{a/b}$ is a QDBDH instance.

## 6 Performance analysis

In this section, we expound a theoretical analysis on the performance of the proposed schemes. Then, we analyze the efficiency of the scheme by numerical simulation. To show the performance more intuitively, we have implemented our scheme, as well as the schemes used by Wu [23] and Wang [24] in the Linux operating system using Pairing-Based Cryptography (PBC) Library [25], programmed in C language, and ran in a virtual machine of a PC (HP PC, 3.1 GHz CPU, and 4 GB RAM). In the experiment, we used elliptical curves with a base field size of 512 bits and an embedding degree of 2. The security levels are selected as |*p*| = 512.

### 6.1 Theoretical analysis

In this section, we compare the computation overhead of the proposed scheme and other schemes from a theoretical perspective. We denote *T*_*e*_, *T*_*p*_, *T*_*h*_, *T*_*H*_, *T*_*mul*_ as the computation cost of exponentiation operation, bilinear pairing operation, general hash function, hash-to-point operation, and multiplication operation, respectively. The running time of those basic operations are presented in Table 1.

**Table 1:** The running time of basic operations (ms).

*T*_*e*_	*T*_*p*_	*T*_*h*_	*T*_*H*_	*T*_*mul*_
3.485	3.724	9.714	0.002	0.017

As shown in Table 1, *T*_*H*_ and *T*_*mul*_ are much smaller than the others, so the hash-to-point operation time and multiplication operation time are negligible. The descending order time of common cryptographic algorithms is *T*_*e*_, *T*_*p*_, *T*_*h*_, *T*_*H*_, *T*_*mul*_, and the computational cost of the bilinear pairing operation is much higher than that in other cryptographic algorithms. The computation cost of the proposed schemes in the index generation and search phases is presented in Table 2. We specify *n* as the number of keywords.

**Table 2:** Comparison of computation cost.

scheme	Index generation	Search
Wu’s scheme [23]	*T*_*e*_ + *T*_*h*_	3*T*_*e*_ + 2*T*_*p*_
Wang’s scheme [24]	(2*n* + 1)*T*_*e*_ + *nT*_*p*_ + *nT*_*h*_	(*n* + 1)*T*_*e*_ + *nT*_*p*_ + *nT*_*h*_
Our scheme	*T*_*e*_ + *T*_*p*_ + *nT*_*h*_	(*n* + 1)*T*_*e*_ + *T*_*p*_ + *nT*_*h*_

As shown in Table 2, in the index generation phase, the descending order of the computation cost is Wang’s scheme [24], our scheme, and Wu’s scheme [23]. In the search phase, the descending order of the computation cost is Wang’s scheme [24], our scheme, and Wu’s scheme [23]. Since Wu’s scheme [23] only implements single-keyword encryption and our scheme implements multi-keyword encryption, the computation cost of our scheme in the index generation and search phase is higher than that of Wu’s scheme [23].

### 6.2 Numerical simulation

We compared our scheme with the schemes proposed by Wu [23] and Wang [24] through numerical simulation. Both our scheme and Wang’s scheme [24] realize multi-keyword search function in ciphertext, whereas Wu’s scheme [23] only realizes single-keyword search. In the numerical simulation, we use the same number of keywords in the index generation and search phases, and compare the computational overhead of different keyword quantities in each phase. We specify the number of keywords as *n* = 100, 200, 300, 400, 500, 600, 700, 800, 900, 1000. The experimental result is the average time for the algorithm to run 10 times. For more information, see S1 Appendix and S1 File.

**Fig 2:**
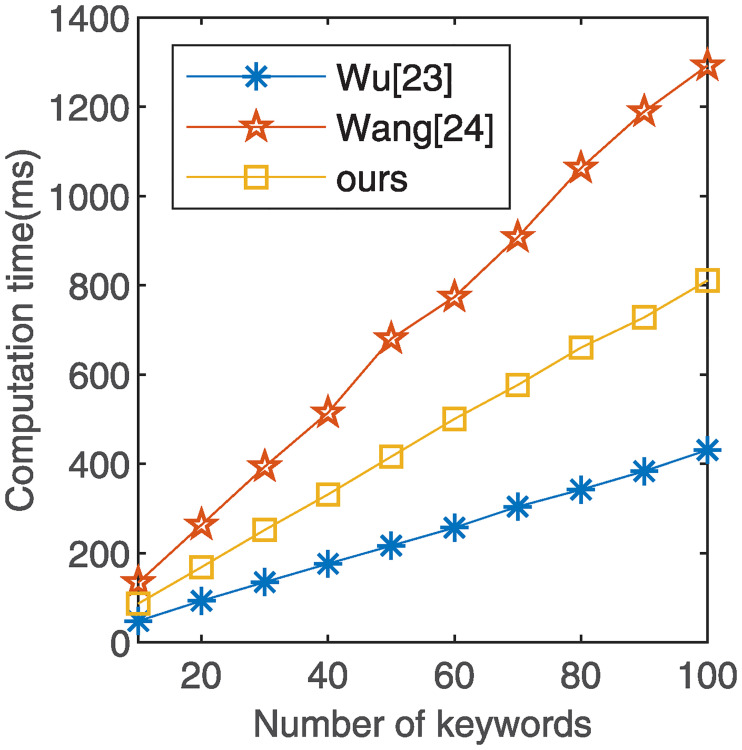
The performance comparison of index generation.

**Fig 3:**
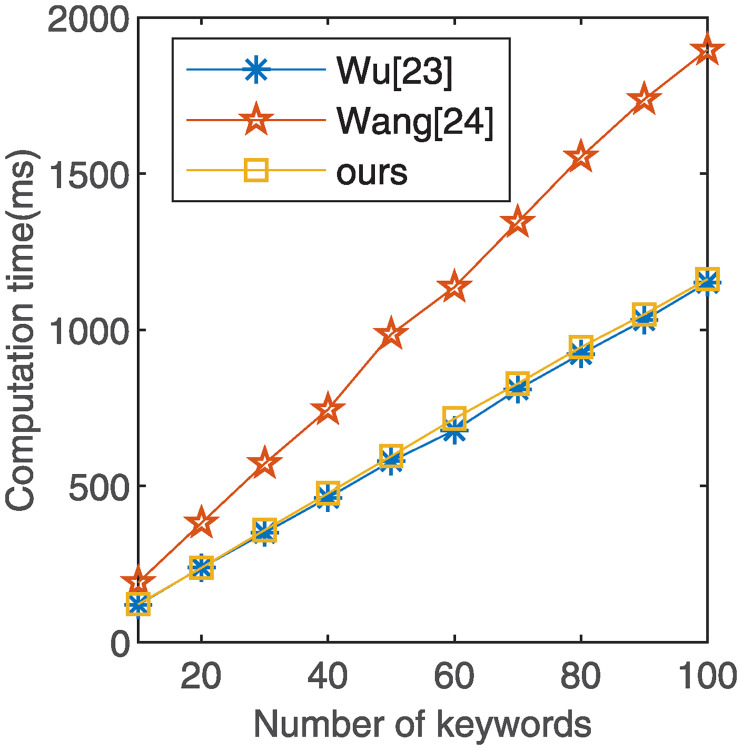
The performance comparison of search.

As illustrated in Fig 2, index generation time increases with the number of keywords. The index generation time of our scheme is less than that of Wang’s scheme [24] but higher than that of Wu’s scheme [23]. The reason is that our scheme uses bilinear pairing operations in the keyword encryption process, but Wu’s scheme [23] is not used. In Fig 3, we present the time cost of the search phase in all schemes. The time spent linearly increases with the number of keywords. Wu’s scheme [23] and our scheme have a subtle difference in the search phase, and both are higher than Wang’s scheme [24].

## 7 Conclusion

We presented an EMR data sharing scheme with privacy protection, secure storage, and secure sharing based on searchable encryption and proxy re-encryption technology, which solves the security problems of data security and personal privacy in the process of EMR sharing based on cloud storage. While protecting the privacy of the patient, this scheme enables patients to access their own EMR. After authorization is provided by the patient, the data users can also access the EMR, which is a practical approach. The EMR ciphertext and keyword index are stored in the cloud server to enable the patient to search EMR with keyword search. The cloud server generates a re-encryption key for the data user after the patient authorizes the data user to access his/her EMR. Then, the cloud server re-encrypts the EMR ciphertext with the re-encryption key and sends it to the data user, who can decrypt it using the private key.

## Supporting information

S1 Appendix Data used to build graphs. The experimental data used for plotting in Figs 2 and 3.
(DOCX)

S1 File Procedure source code. The procedure source code for the numerical simulation of our scheme, Wu’s scheme and Wang’s scheme.
(ZIP)
