# Supplementary material for: A data-sharing scheme that supports multi-keyword search for electronic medical records
Source: PLoS One. 2021 Jan 7;16(1):e0244979. doi: 10.1371/journal.pone.0244979 (PMC7790426; doi:10.1371/journal.pone.0244979)
Supplement: S1 Appendix — The experimental data used for plotting in Figs 2 and 3. (DOCX) [file pone.0244979.s001.docx]

We denote n as the Number of keywords.

We denote t as the Computation time.

Values used to build Fig 2.

Our scheme

| n | 10 | 20 | 30 | 40 | 50 | 60 | 70 | 80 | 90 | 100 |
| --- | --- | --- | --- | --- | --- | --- | --- | --- | --- | --- |
| t | 86.6 | 168.4 | 251.7 | 331.2 | 415.9 | 500.4 | 576.4 | 660.2 | 727.9 | 810.6 |

Wu’s scheme

| n | 10 | 20 | 30 | 40 | 50 | 60 | 70 | 80 | 90 | 100 |
| --- | --- | --- | --- | --- | --- | --- | --- | --- | --- | --- |
| t | 47.5 | 92.9 | 134.7 | 175.9 | 216.3 | 256.7 | 303.8 | 341.9 | 383.3 | 430.7 |

Wang’s scheme

| n | 10 | 20 | 30 | 40 | 50 | 60 | 70 | 80 | 90 | 100 |
| --- | --- | --- | --- | --- | --- | --- | --- | --- | --- | --- |
| t | 133.8 | 263.2 | 393.5 | 514.3 | 680.2 | 773.7 | 907.7 | 1062.7 | 1189.2 | 1292.1 |

Values used to build Fig 3.

Our scheme

| n | 10 | 20 | 30 | 40 | 50 | 60 | 70 | 80 | 90 | 100 |
| --- | --- | --- | --- | --- | --- | --- | --- | --- | --- | --- |
| t | 121.9 | 238.5 | 359.2 | 476.6 | 595.6 | 716.1 | 827.9 | 944.5 | 1049.6 | 1162.3 |

Wu’s scheme

| n | 10 | 20 | 30 | 40 | 50 | 60 | 70 | 80 | 90 | 100 |
| --- | --- | --- | --- | --- | --- | --- | --- | --- | --- | --- |
| t | 120.2 | 239.1 | 350.5 | 462.7 | 579.6 | 678.5 | 809.3 | 922.8 | 1031.3 | 1151.7 |

Wang’s scheme

| n | 10 | 20 | 30 | 40 | 50 | 60 | 70 | 80 | 90 | 100 |
| --- | --- | --- | --- | --- | --- | --- | --- | --- | --- | --- |
| t | 191.6 | 382.1 | 572.3 | 745.1 | 986.1 | 1136.9 | 1344.5 | 1553.5 | 1737.8 | 1896.4 |
