# Supplementary material for: A data-sharing scheme that supports multi-keyword search for electronic medical records
Source: PLoS One. 2021 Jan 7;16(1):e0244979. doi: 10.1371/journal.pone.0244979 (PMC7790426; doi:10.1371/journal.pone.0244979)
Supplement: S1 File — The procedure source code for the numerical simulation of our scheme, Wu’s scheme and Wang’s scheme. (ZIP) [file pone.0244979.s002.zip › S1_File/Code of our scheme.docx]

#include <stdio.h>

#include <pbc.h>

#include <malloc.h>

#include <pbc_test.h>

#include <stdlib.h>

#include <ctype.h>

pairing_t pairing;

int main(int argc, char **argv){

pbc_demo_pairing_init(pairing, argc, argv);

element_t g,Z,p,d,r,pkP,pkD,pkR;

element_t e,B,B1,s,tao,S;

element_t M,k,zk,c1,c2;

element_t *w;

element_t *h;

element_t t1,t2,t;

element_t a,b;

element_t *h2,*T;

element_t T1,e1,e2;

double time1,time2;

//initialize Zr

element_init_Zr(p,pairing);

element_init_Zr(d,pairing);

element_init_Zr(r,pairing);

element_init_Zr(B,pairing);

element_init_Zr(s,pairing);

element_init_Zr(B1,pairing);

element_init_Zr(k,pairing);

element_init_Zr(a,pairing);

element_init_Zr(b,pairing);

//initialize G1

element_init_G1(g,pairing);

element_init_G1(pkP,pairing);

element_init_G1(pkD,pairing);

element_init_G1(pkR,pairing);

element_init_G1(S,pairing);

element_init_G1(tao,pairing);

element_init_G1(c2,pairing);

element_init_G1(t1,pairing);

element_init_G1(e,pairing);

element_init_G1(T1,pairing);

//initialize GT

element_init_GT(Z,pairing);

element_init_GT(M,pairing);

element_init_GT(zk,pairing);

element_init_GT(c1,pairing);

element_init_GT(t2,pairing);

element_init_GT(t,pairing);

element_init_GT(e1,pairing);

element_init_GT(e2,pairing);

printf("Initialization\n");

element_random(g);//生成元g

pairing_apply(Z,g,g,pairing);//Z=e(g,g)

element_random(p);//私钥p

element_random(d);//私钥d

element_random(r);//私钥r

element_pow_zn(pkP,g,p);//公钥P=g^p

element_pow_zn(pkD,g,d);//公钥D=g^d

element_pow_zn(pkR,g,r);//公钥R=g^r

element_random(B);

int n=element_length_in_bytes(B);

element_invert(B1,B);//B1=1/B

/*element_printf("e =%B\n",e);

element_printf("B =%B\n",B);

element_printf("B1 =%B\n",B1);

element_printf("B2 =%B\n",B2);*/

element_pow_zn(tao,g,B1);//tao=g^(1/B)

element_random(s);

element_pow_zn(S,g,s);//S=g^s

int vect_j;

int j;

printf("input the j th:\n");

scanf("%d",&vect_j);

//数组初始化

w = (element_t *)malloc(sizeof(element_t)*vect_j);

h = (element_t *)malloc(sizeof(element_t)*vect_j);

h2 = (element_t *)malloc(sizeof(element_t)*vect_j);

T = (element_t *)malloc(sizeof(element_t)*vect_j);

for(j=0;j<vect_j;j++){

element_init_Zr(w[j],pairing);

element_init_G1(h[j],pairing);

element_init_G1(h2[j],pairing);

element_init_G1(T[j],pairing);

element_random(w[j]);//随机生成关键字w数组

}

/*//输出w

for(j=0;j<vect_j;j++){

element_printf("w= %B\n",w[j]);

}*/

printf("Data Processing\n");

element_random(M);

element_random(k);

element_pow_zn(zk,Z,k);

element_mul(c1,M,zk);

element_mul(c2,pkP,k);

/*element_printf("M= %B\n",M);

element_printf("k= %B\n",k);

element_printf("zk= %B\n",zk);

element_printf("c1= %B\n",c1);

element_printf("c2= %B\n",c2);*/

time1 = pbc_get_time();

for(j=0;j<vect_j;j++){

element_from_hash(h[j], w[j], n);//h=H1(w)

}

element_set1(e);//set e=1

element_mul(t1,e,h[0]);

//element_printf("t1= %B\n",t1);

for(j=1;j<vect_j;j++){

element_mul(t1,t1,h[j]);

//element_printf("t1= %B\n",t1);

}

pairing_apply(t2,t1,g,pairing);//t2=e(t1,g)

element_pow_zn(t,t2,k);//t=e(t1,g)^k

time2 = pbc_get_time();

printf("the time of IndexGen phase =%fs\n",time2-time1);

printf("Search\n");

time1 = pbc_get_time();

element_invert(a,p);//a=1/p

element_mul_zn(b,a,B);

for(j=0;j<vect_j;j++){

element_from_hash(h2[j], w[j], n);//h2=H1(w)

element_pow_zn(T[j],h2[j],b);

//element_printf("T= %B\n",T[j]);

}

element_mul(T1,e,h2[0]);

for(j=1;j<vect_j;j++){

element_mul(T1,T1,h2[j]);

//element_printf("T1= %B\n",T1);

}

pairing_apply(e1,c2,T1,pairing);//e1=e(c2,T1)

element_pow_zn(e2,t,B);

pairing_apply(e1,c2,T1,pairing);//e1=e(c2,T1)

element_pow_zn(e2,t,B);

//element_printf("e1= %B\n",e1);

//element_printf("e2= %B\n",e2);

if(!element_cmp(e1,e2)){//判断等式是否成立

printf("1\n");

}

else{

printf("0\n");

}

time2 = pbc_get_time();

printf("the time of Search phase =%fs\n",time2-time1);

return 0;

}
