# Supplementary material for: A data-sharing scheme that supports multi-keyword search for electronic medical records
Source: PLoS One. 2021 Jan 7;16(1):e0244979. doi: 10.1371/journal.pone.0244979 (PMC7790426; doi:10.1371/journal.pone.0244979)
Supplement: S1 File — The procedure source code for the numerical simulation of our scheme, Wu’s scheme and Wang’s scheme. (ZIP) [file pone.0244979.s002.zip › S1_File/Code of Wang's scheme.docx]

#include <stdio.h>

#include <pbc.h>

#include <malloc.h>

#include <pbc_test.h>

#include <stdlib.h>

#include <ctype.h>

pairing_t pairing;

int main(int argc, char **argv){

pbc_demo_pairing_init(pairing, argc, argv);

element_t g,x,y,X,Y,Z;

element_t r,U;

element_t *w,*h,*h2,*h3,*V;

element_t x1,*a,*T,*Ty,*b,*c;

double time1,time2;

//initialize Zr

element_init_Zr(x,pairing);

element_init_Zr(y,pairing);

element_init_Zr(r,pairing);

element_init_Zr(x1,pairing);

element_init_Zr(x1,pairing);

//initialize G1

element_init_G1(g,pairing);

element_init_G1(X,pairing);

element_init_G1(Y,pairing);

element_init_G1(U,pairing);

//initialize GT

element_init_GT(Z,pairing);

int vect_j;

int j;

printf("input the j th:\n");

scanf("%d",&vect_j);

printf("KeyGen\n");

element_random(g);//生成元g

pairing_apply(Z,g,g,pairing);//Z=e(g,g)

element_random(x);//私钥x

element_random(y);//私钥y

element_pow_zn(X,g,x);//公钥X=g^x

element_pow_zn(Y,g,y);//公钥Y=g^y

//数组初始化

w = (element_t *)malloc(sizeof(element_t)*vect_j);

h = (element_t *)malloc(sizeof(element_t)*vect_j);

h2 = (element_t *)malloc(sizeof(element_t)*vect_j);

h3 = (element_t *)malloc(sizeof(element_t)*vect_j);

V = (element_t *)malloc(sizeof(element_t)*vect_j);

a = (element_t *)malloc(sizeof(element_t)*vect_j);

T = (element_t *)malloc(sizeof(element_t)*vect_j);

Ty = (element_t *)malloc(sizeof(element_t)*vect_j);

b = (element_t *)malloc(sizeof(element_t)*vect_j);

c = (element_t *)malloc(sizeof(element_t)*vect_j);

for(j=0;j<vect_j;j++){

element_init_Zr(w[j],pairing);

element_init_G1(h[j],pairing);

element_init_GT(h2[j],pairing);

element_init_GT(h3[j],pairing);

element_init_GT(V[j],pairing);

element_init_G1(a[j],pairing);

element_init_G1(T[j],pairing);

element_init_G1(Ty[j],pairing);

element_init_GT(b[j],pairing);

element_init_Zr(c[j],pairing);

element_random(w[j]);//随机生成关键字w数组

}

printf("SCF-MPEKS\n");

time1 = pbc_get_time();

element_random(r);

element_pow_zn(U,X,r);

int n=element_length_in_bytes(r);

int n1=element_length_in_bytes(Z);

for(j=0;j<vect_j;j++){

element_from_hash(h[j], w[j], n);//h=H1(w)

pairing_apply(h2[j],h[j],Y,pairing);//h2=e(h,Y)

element_pow_zn(h3[j],h2[j],r);//h3=h2^r=e(h,Y)^r

element_from_hash(V[j], h3[j], n1);//V=H2(h3)

}

time2 = pbc_get_time();

printf("the time of SCF-MPEKS phase =%fs\n",time2-time1);

//输出w

/*for(j=0;j<vect_j;j++){

element_printf("w= %B\n",w[j]);

element_printf("h= %B\n",h[j]);

element_printf("V= %B\n",V[j]);

}*/

printf("Search\n");

time1 = pbc_get_time();

element_invert(x1,x);//x1=1/x

for(j=0;j<vect_j;j++){

element_from_hash(a[j], w[j], n);//a=H2(w[j])

element_pow_zn(T[j],a[j],x1);//T=a^x1

element_pow_zn(Ty[j],T[j],y);

pairing_apply(b[j],Ty[j],U,pairing);//b=e(Ty,U)

element_from_hash(c[j], b[j], n1);//c=H2(b)

if(!element_cmp(c[j],V[j])){//判断等式是否成立

//printf("1\n");

}

else{

//printf("0\n");

}

}

time2 = pbc_get_time();

printf("the time of Search phase =%fs\n",time2-time1);

return 0;

}
