# Supplementary material for: A data-sharing scheme that supports multi-keyword search for electronic medical records
Source: PLoS One. 2021 Jan 7;16(1):e0244979. doi: 10.1371/journal.pone.0244979 (PMC7790426; doi:10.1371/journal.pone.0244979)
Supplement: S1 File — The procedure source code for the numerical simulation of our scheme, Wu’s scheme and Wang’s scheme. (ZIP) [file pone.0244979.s002.zip › S1_File/Code of Wu's scheme.docx]

#include <stdio.h>

#include <pbc.h>

#include <malloc.h>

#include <pbc_test.h>

#include <stdlib.h>

#include <ctype.h>

pairing_t pairing;

int main(int argc, char **argv){

pbc_demo_pairing_init(pairing, argc, argv);

element_t skC,skS,skRi,g,pkC,pkS,pkRi,rk;

element_t *w,*h,*h1,*tw;

element_t r,T1,a;

element_t *h2,*h3,*T2,*e1,*e2,*e3;

double time1,time2;

//initialize Zr

element_init_Zr(skC,pairing);

element_init_Zr(skS,pairing);

element_init_Zr(skRi,pairing);

element_init_Zr(r,pairing);

element_init_Zr(a,pairing);

//initialize G1

element_init_G1(g,pairing);

element_init_G1(pkC,pairing);

element_init_G1(pkS,pairing);

element_init_G1(pkRi,pairing);

element_init_G1(rk,pairing);

element_init_G1(T1,pairing);

//initialize GT

//element_init_GT(Z,pairing);

int vect_j;

int j;

printf("input the j th:\n");

scanf("%d",&vect_j);

printf("Initialization\n");

element_random(g);//生成元g

element_random(skC);

element_random(skS);

element_random(skRi);

element_pow_zn(pkC,g,skC);//公钥X=g^x

element_pow_zn(pkS,g,skS);//公钥X=g^x

element_pow_zn(pkRi,g,skRi);//公钥X=g^x

//element_printf("g= %B\n",g);

//数组初始化

w = (element_t *)malloc(sizeof(element_t)*vect_j);

h = (element_t *)malloc(sizeof(element_t)*vect_j);

h1 = (element_t *)malloc(sizeof(element_t)*vect_j);

tw = (element_t *)malloc(sizeof(element_t)*vect_j);

h2 = (element_t *)malloc(sizeof(element_t)*vect_j);

h3 = (element_t *)malloc(sizeof(element_t)*vect_j);

T2 = (element_t *)malloc(sizeof(element_t)*vect_j);

e1 = (element_t *)malloc(sizeof(element_t)*vect_j);

e2 = (element_t *)malloc(sizeof(element_t)*vect_j);

e3 = (element_t *)malloc(sizeof(element_t)*vect_j);

for(j=0;j<vect_j;j++){

element_init_Zr(w[j],pairing);

element_init_Zr(h[j],pairing);

element_init_Zr(h1[j],pairing);

element_init_G1(tw[j],pairing);

element_init_Zr(h2[j],pairing);

element_init_Zr(h3[j],pairing);

element_init_G1(T2[j],pairing);

element_init_GT(e1[j],pairing);

element_init_GT(e2[j],pairing);

element_init_GT(e3[j],pairing);

}

printf("Data processing\n");

time1 = pbc_get_time();

int n=element_length_in_bytes(skC);

for(j=0;j<vect_j;j++){

element_random(w[j]);//随机生成关键字w数组

element_from_hash(h[j], w[j], n);//h=H1(w)

element_mul_zn(h1[j],h[j],skS);

element_pow_zn(tw[j],pkC,h1[j]);

}

element_pow_zn(rk,pkRi,skS);

time2 = pbc_get_time();

printf("the time of IndexGen phase =%fs\n",time2-time1);

printf("Search\n");

time1 = pbc_get_time();

element_random(r);

element_pow_zn(T1,pkC,r);

element_mul_zn(a,r,skRi);

for(j=0;j<vect_j;j++){

element_from_hash(h2[j], w[j], n);//h2=H(w)

element_mul_zn(h3[j],h2[j],a);

element_pow_zn(T2[j],pkC,h3[j]);

pairing_apply(e1[j],tw[j],T1,pairing);//e1=e(tw,T1)

pairing_apply(e2[j],T2[j],rk,pairing);//e2=e(T2,rk)

element_pow_zn(e3[j],e2[j],skC);

if(!element_cmp(e1[j],e3[j])){//判断等式是否成立

printf("1\n");

}

else{

printf("0\n");

}

}

time2 = pbc_get_time();

printf("the time of Search phase =%fs\n",time2-time1);

return 0;

}
